# Montessori education: a review of the evidence base

**DOI:** 10.1038/s41539-017-0012-7

**Published:** 2017-10-27

**Authors:** Chloë Marshall

**Affiliations:** Department of Psychology and Human Development, UCL Institute of Education, University College London, London, UK

## Abstract

The Montessori educational method has existed for over 100 years, but evaluations of its effectiveness are scarce. This review paper has three aims, namely to (1) identify some key elements of the method, (2) review existing evaluations of Montessori education, and (3) review studies that do not explicitly evaluate Montessori education but which evaluate the key elements identified in (1). The goal of the paper is therefore to provide a review of the evidence base for Montessori education, with the dual aspirations of stimulating future research and helping teachers to better understand whether and why Montessori education might be effective.

## Introduction

Maria Montessori (1870–1952) was by any measure an extraordinary individual. She initially resisted going into teaching—one of the few professions available to women in the late 19th century—and instead became one of the very first women to qualify as a medical doctor in Italy. As a doctor she specialised in psychiatry and paediatrics. While working with children with intellectual disabilities she gained the important insight that in order to learn, they required not medical treatment but rather an appropriate pedagogy. In 1900, she was given the opportunity to begin developing her pedagogy when she was appointed director of an Orthophrenic school for developmentally disabled children in Rome. When her pupils did as well in their exams as typically developing pupils and praise was lavished upon her for this achievement, she did not lap up that praise; rather, she wondered what it was about the education system in Italy that was failing children without disabilities. What was holding them back and preventing them from reaching their potential? In 1907 she had the opportunity to start working with non-disabled children in a housing project located in a slum district of Rome. There, she set up her first 'Casa dei Bambini' ('children’s house') for 3–7-year olds. She continued to develop her distinctive pedagogy based on a scientific approach of experimentation and observation. On the basis of this work, she argued that children pass through sensitive periods for learning and several stages of development, and that children’s self-construction can be fostered through engaging with self-directed activities in a specially prepared environment. There was international interest in this new way of teaching, and there are now thousands of Montessori schools (predominantly for children aged 3–6 and 6–12) throughout the world.^1–4^

Central to Montessori’s method of education is the dynamic triad of child, teacher and environment. One of the teacher’s roles is to guide the child through what Montessori termed the 'prepared environment, i.e., a classroom and a way of learning that are designed to support the child’s intellectual, physical, emotional and social development through active exploration, choice and independent learning. One way of making sense of the Montessori method for the purposes of this review is to consider two of its important aspects: the learning materials, and the way in which the teacher and the design of the prepared environment promote children’s self-directed engagement with those materials. With respect to the learning materials, Montessori developed a set of manipulable objects designed to support children’s learning of sensorial concepts such as dimension, colour, shape and texture, and academic concepts of mathematics, literacy, science, geography and history. With respect to engagement, children learn by engaging hands-on with the materials most often individually, but also in pairs or small groups, during a 3-h 'work cycle' in which they are guided by the teacher to choose their own activities. They are given the freedom to choose what they work on, where they work, with whom they work, and for how long they work on any particular activity, all within the limits of the class rules. No competition is set up between children, and there is no system of extrinsic rewards or punishments. These two aspects—the learning materials themselves, and the nature of the learning—make Montessori classrooms look strikingly different to conventional classrooms.

It should be noted that for Montessori the goal of education is to allow the child’s optimal development (intellectual, physical, emotional and social) to unfold.^2^ This is a very different goal to that of most education systems today, where the focus is on attainment in academic subjects such as literacy and mathematics. Thus when we ask the question, as this review paper does, whether children benefit more from a Montessori education than from a non-Montessori education, we need to bear in mind that the outcome measures used to capture effectiveness do not necessarily measure the things that Montessori deemed most important in education. Teachers and parents who choose the Montessori method may choose it for reasons that are not so amenable to evaluation.

Despite its existence for over 100 years, peer-reviewed evaluations of Montessori education are few and they suffer from a number of methodological limitations, as will be discussed in Section 3. This review has three aims, namely to (1) identify some key elements of the Montessori educational method, (2) review existing evaluations of Montessori education, and (3) review studies that do not explicitly evaluate Montessori education but which evaluate the key elements identified in (1). My goal is to provide a review of the scientific evidence base for Montessori education, with the dual aspirations of stimulating future research and helping teachers to better understand whether and why Montessori education might be effective.

## Some key elements of the Montessori educational method

The goal of this section is to isolate some key elements of the Montessori method, in order to better understand why, if Montessori education is effective, this might be, and what elements of it might usefully be evaluated by researchers. These are important considerations because there is considerable variability in how the Montessori method is implemented in different schools, and the name, which is not copyrighted, is frequently used without full adherence.^5,6^ Nevertheless, some elements of the method might still be beneficial, or could be successfully incorporated (or, indeed, are already incorporated) into schools that do not want to carry the name 'Montessori' or to adhere fully to its principles. Pinpointing more precisely what—if anything—about the Montessori method is effective will enable a better understanding of why it works. Furthermore, it has been argued that there might be dangers in adopting wholesale and uncritically an educational method that originated over 100 years ago, in a world that was different in many ways to today’s.^7^ If the method is to be adopted piecemeal, which pieces should be adopted? As outlined previously, two important aspects of Montessori’s educational method are the learning materials, and the self-directed nature of children’s engagement with those materials. Some key elements of each of these aspects will now be considered in turn.

### The learning materials

The first learning materials that the child is likely to encounter in the Montessori classroom are those that make up the practical life curriculum. These are activities that involve pouring different materials, using utensils such as scissors, tongs and tweezers, cleaning and polishing, preparing snacks, laying the table and washing dishes, arranging flowers, gardening, doing up and undoing clothes fastenings, and so on. Their aims, in addition to developing the child’s skills for independent living, are to build up the child’s gross and fine motor control and eye-hand co-ordination, to introduce them to the cycle of selecting, initiating, completing and tidying up an activity (of which more in the next section), and to introduce the rules for functioning in the social setting of the classroom.

As the child settles into the cycle of work and shows the ability to focus on self-selected activities, the teacher will introduce the sensorial materials. The key feature of the sensorial materials is that each isolates just one concept for the child to focus on. The pink tower, for example, consists of ten cubes which differ only in their dimensions, the smallest being 1 cm^3^, the largest 10 cm^3^. In building the tower the child’s attention is being focused solely on the regular decrease in volume of successive cubes. There are no additional cues—different colours for example, or numbers written onto the faces of the cube—which might help the child to sequence the cubes accurately. Another piece of sensorial material, the sound boxes, contains six pairs of closed cylinders that vary in sound from soft to loud when shaken, and the task for the child is to find the matching pairs. Again, there is only one cue that the child can use to do this task: sound. The aim of the sensorial materials is not to bombard the child’s senses with stimuli; on the contrary, they are tools designed for enabling the child to classify and put names to the stimuli that he will encounter on an everyday basis.

The sensorial materials, are, furthermore, designed as preparation for academic subjects. The long rods, which comprise ten red rods varying solely in length in 10 cm increments from 10 cm to 1 m, have an equivalent in the mathematics materials: the number rods, where the rods are divided into alternating 10 cm sections of red and blue so that they take on the numerical values 1–10. The touchboards, which consist of alternate strips of sandpaper and smooth paper for the child to feel, are preparation for the sandpaper globe in geography—a globe where the land masses are made of rough sandpaper but the oceans and seas are smooth. The touchboards are also preparation for the sandpaper letters in literacy and sandpaper numerals in mathematics, which the child learns to trace with his index and middle fingers.

Key elements of the literacy curriculum include the introduction of writing before reading, the breaking down of the constituent skills of writing (pencil control, letter formation, spelling) before the child actually writes words on paper, and the use of phonics for teaching sound-letter correspondences. Grammar—parts of speech, morphology, sentence structure—are taught systematically through teacher and child-made materials.

In the mathematics curriculum, quantities 0–10 and their symbols are introduced separately before being combined, and large quantities and symbols (tens, hundreds and thousands) and fractions are introduced soon after, all through concrete materials. Operations (addition, subtraction, multiplication, division, the calculation of square roots) are again introduced using concrete materials, which the child can choose to stop using when he is able to succeed without that concrete support.

Principles running throughout the design of these learning materials are that the child learns through movement and gains a concrete foundation with the aim of preparing him for learning more abstract concepts. A further design principle is that each piece of learning material has a 'control of error' which alerts the child to any mistakes, thereby allowing self-correction with minimal teacher support.

### Self-directed engagement with the materials

Important though the learning materials are,^8^ they do not, in isolation, constitute the Montessori method because they need to be engaged with in a particular way. Montessori observed that the young child is capable of concentrating for long periods of time on activities that capture his spontaneous interest.^2–4^ There are two features of the way that children engage with the learning materials that Montessori claimed promoted this concentration. The first is that there is a cycle of activity surrounding the use of each piece of material (termed the 'internal work cycle^'9^). If a child wishes to use the pink tower, for example, he will have to find a space on the floor large enough to unroll the mat that will delineate his work area, carry the ten cubes of the pink tower individually to the mat from where they are stored, then build the tower. Once he has built the tower he is free to repeat this activity as many times as he likes. Other children may come and watch, and if he wishes they can join in with him, but he will be able to continue on his own if he prefers and for as long as he likes. When he has had enough, he will dismantle the pink tower and reassemble it in its original location, ready for another child to use. This repeated and self-chosen engagement with the material, the lack of interruption, and the requirement to set up the material and put it away afterwards, are key elements aimed at developing the child’s concentration.^10^

The second feature which aims to promote concentration is that these cycles of activity take place during a 3-h period of time (termed the 'external work cycle'^9^). During those 3 h children are mostly free to select activities on their own and with others, and to find their own rhythm of activity, moving freely around the classroom as they do so. One might wonder what the role of the teacher is during this period. Although the children have a great deal of freedom in what they do, their freedom is not unlimited. The teacher’s role is to guide children who are finding it hard to select materials or who are disturbing others, to introduce new materials to children who are ready for a new challenge, and to conduct small-group lessons. Her decisions about what to teach are made on the basis of careful observations of the children. Although she might start the day with plans of what she will do during the work cycle, she will be led by her students and their needs, and there is no formal timetable. Hence the Montessori classroom is very different to the teacher-led conventional classroom with its highly structured day where short timeslots are devoted to each activity, the whole class is engaged in the same activities at the same time, and the teacher instructs at the front of the class.

In summary, there are two aspects of Montessori classrooms that are very different to conventional classrooms: the learning materials themselves, and the individual, self-directed nature of the learning under the teacher’s expert guidance. All the elements described here—the features of the learning materials themselves (e.g., each piece of material isolates just one concept, each contains a control of error that allows for self-correction, learning proceeds from concrete to abstract concepts) and the child-led manner of engagement with those materials (e.g., self-selection, repeated and active engagement, tidying up afterwards, freedom from interruption, lack of grades and extrinsic rewards) might potentially benefit development and learning over the teaching of the conventional classroom. We will return to many of the elements discussed here in the following two sections. (This has necessarily been only a brief survey of some of the most important elements of the Montessori method. Readers wanting to find out more are again directed to refs. 2–4).

## Evaluations of Montessori education

There are few peer-reviewed evaluations of Montessori education, and the majority have been carried out in the USA. Some have evaluated children’s outcomes while those children were in Montessori settings, and others have evaluated Montessori-educated children after a period of subsequent conventional schooling. As a whole this body of research suffers from several methodological limitations. Firstly, few studies are longitudinal in design. Secondly, there are no good quality randomised control trials; most researchers have instead tried to match participants in Montessori and comparison groups on as many likely confounding variables as possible. Thirdly, if children in the Montessori group do score higher than those in the non-Montessori group on a particular outcome measure, then assuming that that effect can be attributed to being in a Montessori classroom, what exactly is it about Montessori education that has caused the effect? Montessori education is a complex package—how can the specific elements which might be causing the effect be isolated? At a very basic level—and drawing on two of the main aspects of Montessori education outlined above—is the effect due to the learning materials or to the self-directed way in which children engage with them (and can the two be separated)? Fourthly, there are presumably differences between Montessori schools (including the way in which the method is implemented) that might influence children’s outcomes, but studies rarely include more than one Montessori school, and sometimes not more than one Montessori class. Fifthly, and relatedly, there is the issue of 'treatment fidelity'—what counts as a Montessori classroom? Not all schools that call themselves 'Montessori' adhere strictly to Montessori principles, have trained Montessori teachers, or are accredited by a professional organisation. A sixth, and again related, point is that children’s experiences in Montessori education will vary in terms of the length of time they spend in Montessori education, and the age at which they attend. Finally, the numbers of children participating in studies are usually small and quite narrow in terms of their demographics, making generalisation of any results problematic. These methodological issues are not limited to evaluations of Montessori education, of course—they are relevant to much of educational research.

Of these, the lack of randomised control trials is particularly notable given the recognition of their importance in education.^11,12^ Parents choose their child’s school for a host of different reasons,^13^ and randomisation is important in the context of Montessori education because parents who choose a non-conventional school for their child might be different in relevant ways from parents who do not, for example in their views on child-rearing and aspirations for their child’s future. This means that if a study finds a benefit for Montessori education over conventional education this might reflect a parent effect rather than a school effect. Furthermore, randomisation also controls for socio-economic status (SES). Montessori schools are often fee-paying, which means that pupils are likely to come from higher SES families; children from higher SES families are likely to do better in a variety of educational contexts.^14–16^ A recent report found that even public (i.e., non-fee-paying) Montessori schools in the USA are not representative of the racial and socioeconomic diversity of the neighbourhoods they serve.^17^ However, random assignment of children to Montessori versus non-Montessori schools for the purposes of a randomised control trial would be very difficult to achieve because it would take away parental choice.

Arguably the most robust evaluation of the Montessori method to date is that by Lillard and Else-Quest.^18^ They compared children in Montessori and non-Montessori education and from two age groups—5 and 12-year olds—on a range of cognitive, academic, social and behavioural measures. Careful thought was given to how to overcome the lack of random assignment to the Montessori and non-Montessori groups. The authors’ solution was to design their study around the school lottery that was already in place in that particular school district. All children had entered the Montessori school lottery; those who were accepted were assigned to the Montessori group, and those who were not accepted were assigned to the comparison (other education systems) group. Post-hoc comparisons showed similar income levels in both sets of families. Although group differences were not found for all outcome measures, where they were found they favoured the Montessori group. For 5-year olds, significant group differences were found for certain academic skills (namely letter-word identification, phonological decoding ability, and math skills), a measure of executive function (the card sort task), social skills (as measured by social reasoning and positive shared play) and theory of mind (as measured by a false-belief task). For 12-year olds, significant group differences were found on measures of story writing and social skills. Furthermore, in a questionnaire that asked about how they felt about school, responses of children in the Montessori group indicated that they felt a greater sense of community. The authors concluded that 'at least when strictly implemented, Montessori education fosters social and academic skills that are equal or superior to those fostered by a pool of other types of schools'.^18^

Their study has been criticised for using just one Montessori school,^19^ but Lillard and Else-Quest’s response is that the school was faithful to Montessori principles, which suggests that the results might be generalisable to other such schools.^20^ That fidelity might impact outcomes has long been of concern,^21^ and was demonstrated empirically in a further, longitudinal, study,^6^ that compared high fidelity Montessori classes (again, from just one school), 'supplemented' Montessori classes (which provided the Montessori materials plus conventional activities such as puzzles, games and worksheets), and conventional classrooms. Children in these classes were 3–6 years old, and they were tested at two time-points: towards the beginning and towards the end of the school year. Although the study lacked random assignment of children to groups, the groups were matched with respect to key parent variables such as parental education. As in Lillard and Else-Quest’s earlier study,^18^ outcome measures tapped a range of social and academic skills related to school readiness (i.e., children’s preparedness to succeed in academic settings). There were two research questions: firstly, do preschool children’s school readiness skills change during the academic year as a function of school type, and secondly, within Montessori schools, does the percentage of children using Montessori materials in a classroom predict children’s school readiness skills at the end of the academic year? Overall, the answer to both questions was “yes”. Children in the high-fidelity Montessori school, as compared with children in the other two types of school, showed significantly greater gains on measures of executive function, reading, math, vocabulary, and social problem-solving. Furthermore, the degree to which children were engaged with Montessori materials significantly predicted gains in executive function, reading and vocabulary. In other words, treatment fidelity mattered: children gained fewer benefits from being in a Montessori school when they were engaged in non-Montessori activities.

This study does not demonstrate definitively that the Montessori materials drove the effect: there might have been other differences between the high and lower fidelity classrooms—such as the teachers’ interactions with their pupils—that were responsible for the difference in child outcomes.^6^ In a move to explore the role of the Montessori materials further, a more recent experimental study^22^ removed supplementary materials, to leave just the Montessori materials, from two of the three classrooms in a Montessori school that served 3–6-year olds. Over a period of 4 months children in the classrooms from which supplementary materials were removed made significantly greater gains than children from the unchanged classroom on tests of letter-word identification and executive function, although not on measures of vocabulary, theory of mind, maths, or social problem-solving. The authors acknowledge weaknesses in the study design, including the small number of participants (just 52 across the three classrooms) and the short duration. Nevertheless, the study does provide a template for how future experimental manipulations of fidelity to the Montessori method could be carried out.

Fidelity is important because variation in how faithful Montessori schools are to the 'ideal' is likely to be an important factor in explaining why such mixed findings have been found in evaluations of the Montessori method.^6^ For example, two early randomised control trials to evaluate Head Start in the USA did not find any immediate benefit of Montessori preschool programmes over other types of preschool programmes.^23,24^ In both programmes, only 4-year olds were included, whereas the ideal in Montessori preschool programmes is for 3–6 year olds to be taught in the same class in order to foster child-to-child tutoring.^6^ Furthermore, in one of the programmes^23^ the ideal 3-h work cycle was reduced to just 30 min.^6^ A more recent study of older children compared 8th grade Montessori and non-Montessori students matched for gender, ethnicity and socio-economic status.^25^ The study found lower scores for Montessori students for English/Language Arts and no difference for maths scores, but the participating Montessori school altered the “ideal” by issuing evaluative grades to pupils and introducing non-Montessori activities.^6^

These same limitations then make it difficult to interpret studies that have found 'later' benefits for children who have been followed up after a subsequent period of conventional education. In one of the studies discussed earlier,^23^ social and cognitive benefits did emerge for children who had previously attended Montessori preschools and then moved to conventional schools, but these benefits did not emerge until adolescence, while a follow-up study^26^ found cognitive benefits in Montessori males only, again in adolescence. Although such 'sleeper effects' have been widely reported in evaluations of early years interventions, they may be artefacts of simple measurement error and random fluctuations.^27^ Importantly, if the argument is that lack of fidelity to the Montessori method is responsible for studies not finding significant benefits of Montessori education at younger ages, it is not logical to then credit the Montessori method with any benefits that emerge in follow-up studies.

Some studies report positive outcomes for certain curricular areas but not others. One, for example, investigated scores on maths, science, English and social studies tests in the final years of compulsory education, several years after children had left their Montessori classrooms.^28^ Compared to the non-Montessori group (who were matched for gender, socioeconomic status, race/ethnicity and high school attended), the Montessori group scored significantly higher on maths and science, but no differences were found for English and social studies. What might explain this differential effect? The authors suggested that the advantages for maths might be driven by the materials themselves, compared to how maths is taught in conventional classes.^28^ Alternatively, or perhaps in addition, children in Montessori classrooms might spend more time engaged in maths and science activities compared to children in conventional classes, with the amount of time spent on English and social studies not differing. However, the authors were unable, within the design of their study, to provide details of exactly how much time children in the Montessori school had spent doing maths, science, English and social studies, in comparison to the time that children in conventional classes were spending on those subjects.

Just as knowing what is going on in the Montessori classroom is vital to being able to interpret the findings of evaluations, so is knowing what is going on in the comparison classrooms. One of the earliest evaluations of Montessori education in the USA^29^ speculated that Montessori would have found much to appreciate in one of the non-Montessori comparison classes, including its 'freedom for the children (moving about; working alone); its planned environment (innovative methods with tape recorder playback of children’s conversations; live animals, etc.); its non-punitive character (an “incorrect” answer deserves help, not anger; original answers are reinforced, but other answers are pursued); and its emphasis on concentration (the children sustained activity without direct supervision for relatively long periods of time)'. In some evaluations, the differences between Montessori and conventional classrooms might not actually be so great, which might explain why benefits of being educated in a Montessori classroom are not found. And even if the Montessori approach to teaching a particular curriculum area is different to those used in conventional classrooms, there are likely to be different, equally-effective approaches to teaching the same concepts. This is a suggested explanation for the finding that although children in Montessori kindergartens had an advantage relative to their conventionally-educated peers for base-10 understanding in mathematics, they did not maintain this advantage when tested 2 years later.^30^

While most evaluations are interested in traditional academic outcomes or factors related to academic success such as executive functions, a small number have investigated creativity. For example, an old study^31^ compared just 14 four and five-year-old children who attended a Montessori nursery school with 14 four and five-year olds who attended a conventional nursery school (matched for a range of parental variables, including attitudes and parental control). In a non-verbal creativity task, involving picture construction, they were given a blank sheet of paper, a piece of red gummed paper in the shape of a curved jellybean, and a pencil. They were then asked to think of and draw a picture in which the red paper would form an integral part. Each child’s construction was rated for originality, elaboration, activity, and title adequacy, and these ratings were then combined into a 'creativity' score. The group of conventionally-schooled children scored almost twice as high as the Montessori group. A second task involved the child giving verbal descriptions of seven objects: a red rubber ball, a green wooden cube, a short length of rope, a steel mirror, a piece of rectangular clear plastic, a piece of chalk, and a short length of plastic tubing. Each description was scored as to whether it was functional (i.e., focused on the object’s use) or whether it was a description of the object’s physical characteristics (i.e., shape, colour, etc.). Like the non-verbal creativity task, this task differentiated the two groups: whereas the conventionally educated children gave more functional descriptions (e.g., for the cube: “you play with it”), the Montessori children gave more physical descriptions (e.g., “it’s square, it’s made of wood, and it’s green”). A third task, the Embedded Figure Test, involved the child first being presented with a stimulus figure and then locating a similar figure located in an embedding context. Both accuracy and speed were measured. While the two groups did not differ in the number of embedded figures accurately located, the Montessori group completed the task significantly more quickly. The fourth and final task required children to draw a picture of anything they wanted to. Drawings were coded for the presence or absence of geometric figures and people. The Montessori group produced more geometric figures, but fewer people, than the conventional group.

The authors were careful not to cast judgement on the performance differences between the two groups.^31^ They wrote that 'The study does, however, support the notion that differing preschool educational environments yield different outcomes' and 'Montessori children responded to the emphasis in their programme upon the physical world and upon a definition of school as a place of work; the Nursery School children responded on their part to the social emphasis and the opportunity for spontaneous expression of feeling'. They did not, however, compare and contrast the particular features of the two educational settings that might have given rise to these differences.

Creativity has been studied more recently in France.^32^ Seven to twelve-year olds were tested longitudinally on five tasks tapping different aspects of creativity. 'Divergent' thinking tasks required children to (1) think of unusual uses for a cardboard box, (2) come up with ideas for making a plain toy elephant more entertaining, and (3) make as many drawings as possible starting from pairs of parallel lines. 'Integrative' thinking tasks required children to (1) invent a story based on a title that was provided to them, and (2) invent a drawing incorporating six particular shapes. Their sample was bigger than that of the previous study,^31^ comprising 40 pupils from a Montessori school and 119 from two conventional schools, and pupils were tested in two consecutive years (no information is provided about whether pupils from different schools were matched on any variable other than age). For both types of task and at both time-points the Montessori-educated children scored higher than the conventionally-educated children. Again, the authors made little attempt to pinpoint the precise differences between schools that might have caused such differences in performance.

None of the studies discussed so far has attempted to isolate individual elements of the Montessori method that might be accounting for any of the positive effects that they find. There are several studies, however, that have focused on the practical life materials. A quasi-experimental study^33^ demonstrated that the practical life materials can be efficacious in non-Montessori classrooms. More than 50 different practical life exercises were introduced into eight conventional kindergarten classes, while five conventional kindergarten classes were not given these materials and acted as a comparison group. The outcome measure was a fine motor control task, the 'penny posting test', whereby the number of pennies that a child could pick up and post through a one-inch slot in a can in two 30 s trials was counted. At pre-test the treatment and comparison groups did not differ in the number of pennies posted, but at post-test 6 months later the treatment group achieved a higher score than the comparison group, indicating finer motor control. A nice feature of this study is that teachers reported children in both groups spending the same amount of time on tasks designed to support fine motor control development, suggesting that there was something specific to the design of the practical life materials that was more effective in this regard than the conventional kindergarten materials on offer. And because the preschools that had used the practical life activities had introduced no other elements of the Montessori method, the effect could be confidently attributed to the practical life materials themselves.

An extension of this study^34^ investigated the potential benefits of the practical life materials for fine motor control by comparing 5-year olds in Montessori kindergarten programmes with 5-year olds in a conventional programme (reported to have similarities in teaching mission and pupil background characteristics) on the 'flag posting test'. In this task, the child was given a solid hardwood tray covered with clay in which there were 12 pinholes. There were also 12 paper flags mounted on pins, six to the right of the tray and six to the left, and the child’s task was to place the flags one at time in the holes. The child received three scores: one for the amount of time taken to finish the activity, one for the number of attempts it took the child to put each flag into the hole, and one for hand dominance (to receive a score of 1 (established dominance) the child had to consistently use the same hand to place all 12 flags, whereas mixed dominance received a score of 0). Children were pre-tested at the beginning of the school year and post-tested 8 months later. Despite the lack of random assignment to groups, the two groups did not differ on pre-test scores, but they did at post-test: at post-test the Montessori group were significantly faster and significantly more accurate at the task, and had more established hand dominance. However, no attempt was made to measure how frequently children in both groups engaged with materials and activities that were designed to support fine motor control development. Furthermore, the children in the Montessori classrooms were at the age where they should also have been using the sensorial materials, some of which (for example, the 'knobbed cylinders' and 'geometric cabinet') are manipulated by holding small knobs, and whose use could potentially enhance fine motor control. At that age children would also have been using the 'insets for design', materials from the early literacy curriculum designed to enhance pencil control. Therefore, although the results of this study are consistent with the practical life materials enhancing fine motor control, the study does not securely establish that they do.

A further study^35^ introduced practical life exercises into conventional kindergarten classes, while control kindergarten classes were not given these materials. 15 min were set aside in the experimental schools’ timetable for using the practical life materials, and they were also available during free choice periods. This time the outcome measure at pre-test and post-test was not fine motor skill but attention. There were benefits to attention of being in the experimental group, but only for girls—boys showed no such benefits. The differential gender impact of the practical life materials on the development of attention is puzzling. Girls did not appear to engage with the materials more than boys during the time that was set aside for using them, but no measure was taken of whether girls chose them more frequently than boys during the free choice periods. Similarly, there were no measurements of the time that children in both the experimental and control groups spent engaged in other activities that might have enhanced fine motor control. Nor is it clear whether it was the fine motor practice directly or rather the opportunity to select interesting activities (the teachers in the experimental schools commented on how interesting the children found the practical life activities) that was responsible for the benefits to attention that were recorded for girls.

Finally, it has been found that young adolescents in Montessori middle schools show greater intrinsic motivation than their peers in conventional middle schools (matched for an impressive array of background variables, including ethnicity, parental education and employment, home resources, parental involvement in school, and number of siblings).^36^ The authors did not establish exactly which elements of the Montessori method might be responsible for this finding, but they did speculate that the following might be relevant: “students were provided at least 2 h per day to exercise choice and self-regulation; none of the students received mandatory grades; student grouping was primarily based on shared interests, not standardised tests; and students collaborated often with other students”. The authors did not evaluate the Montessori and non-Montessori groups on any measures of academic outcomes, but given the links between academic success and motivation at all stages of education (they provide a useful review of this literature), this link would be worth investigating in Montessori schools.

This section has discussed studies that have evaluated the Montessori method directly. To date there have been very few methodologically robust evaluations. Many suffer from limitations that make it challenging to interpret their findings, whether those findings are favourable, neutral or unfavourable towards the Montessori method. However, while randomised control trials could (and should) be designed to evaluate individual elements of the Montessori method, it is difficult to see how the random assignment of pupils to schools could work in practice (hence the ingenuity of the study reported in ref. 18). Nor could trials be appropriately blinded—teachers, and perhaps parents and pupils too, would know whether they were in the Montessori arm of the trial. In other words, although random assignment and blinding might work for specific interventions, it is hard to see how they could work for an entire school curriculum. Furthermore, given the complexity of identifying what it is that works, why it works, and for whom it works best, additional information, for example from observations of what children and teachers are actually doing in the classroom, would be needed for interpreting the results.

## Evaluations of key elements of Montessori education that are shared with other educational methods

This final section examines studies that have not evaluated the Montessori method directly, but have evaluated other educational methods and interventions that share elements of the Montessori method. They, together with our growing understanding of the science underpinning learning, can add to the evidence base for Montessori education. Given the vast amount of research and the limited space in which to consider it, priority is given to systematic reviews and meta-analyses.

One of the best-researched instructional techniques is the use of phonics for teaching children to read. Phonics is the explicit teaching of the letter-sound correspondences that allow the child to crack the alphabetic code. Montessori’s first schools were in Italy, and Italian orthography has relatively transparent one-to-one mappings between letters and sounds, making phonics a logical choice of method for teaching children the mechanics of reading and spelling. English orthography is, however, much less regular: the mappings between letters and sounds are many-to-many, and for this reason the use of phonics as a method of instruction has been challenged for English.^37^ Nevertheless, there is overwhelming evidence of its effectiveness despite English’s irregularities.^38–40^ At the same time, great strides have been made in elucidating the neural mechanisms that underlie early reading and reading impairments, and these too demonstrate the importance to successful reading of integrating sound and visual representations.^41^

As always in education, the devil is in the detail. Importantly, phonics programmes have the greatest impact on reading accuracy when they are systematic.^39,40^ By 'systematic' it is meant that letter-sound relationships are taught in an organised sequence, rather than being taught on an ad hoc as-and-when-needed basis. However, within systematic teaching of phonics there are two very different approaches: synthetic phonics and analytic phonics. Synthetic phonics starts from the parts and works up to the whole: children learn the sounds that correspond to letters or groups of letters and use this knowledge to sound out words from left to right. Analytic phonics starts from the whole and drills down to the parts: sound-letter relationships are inferred from sets of words which share a letter and sound, e.g., $\underline{h}$ *at*, $\underline{h}$ *en*, $\underline{h}$ *ill*, $\underline{h}$ *orse*. Few randomised control trials have pitted synthetic and analytic phonics against one another, and it is not clear that either has the advantage.^40^

The Montessori approach to teaching phonics is certainly systematic. Many schools in the UK, for example, use word lists drawn from Morris’s 'Phonics 44'.^42,43^ Furthermore, the Montessori approach to phonics is synthetic rather than analytic: children are taught the sound-letter code before using it to encode words (in spelling) and decode them (in reading). One of the criticisms of synthetic phonics is that it teaches letters and sounds removed from their meaningful language context, in a way that analytic phonics does not.^44^ It has long been recognised that the goal of reading is comprehension. Reading for meaning requires both code-based skills and language skills such as vocabulary, morphology, syntax and inferencing skills,^45^ and these two sets of skills are not rigidly separated, but rather interact at multiple levels.^46^ Indeed, phonics instruction works best where it is integrated with text-level reading instruction.^39,40^ The explicit teaching of phonics within a rich language context—both spoken and written—is central to the Montessori curriculum. No evaluations have yet pitted phonics teaching in the Montessori classroom versus phonics teaching in the conventional classroom, however, and so whether the former is differentially effective is not known.

Research into writing supports Montessori’s view that writing involves a multitude of component skills, including handwriting, spelling, vocabulary and sentence construction.^47,48^ Proficiency in these skills predicts the quality of children’s written compositions.^49,50^ In the Montessori classroom these skills are worked on independently before being brought together, but they can continue to be practised independently. A growing body of research from conventional and special education classrooms demonstrates that the specific teaching of the component skills of writing improves the quality of children’s written compositions.^51–54^

With respect to teaching mathematics to young children, there are many recommendations that Montessori teachers would recognise in their own classrooms, such as teaching geometry, number and operations using a developmental progression, and using progress monitoring to ensure that mathematics instruction builds on what each child knows.^55^ Some of the recommended activities, such as 'help children to recognise, name, and compare shapes, and then teach them to combine and separate shapes'^55^ map exactly on to Montessori’s sensorial materials such as the geometric cabinet and the constructive triangles. Other activities such as 'encourage children to label collections with number words and numerals'^55^ map onto Montessori’s early mathematics material such as the number rods, the spindle box and the cards and counters. The importance of conceptual knowledge as the foundation for children being able to understand fractions has been stressed.^56^ The Montessori fraction circles—which provide a sensorial experience with the fractions from one whole to ten tenths—provide just such a foundation, as do practical life exercises such as preparing snacks (how should a banana be cut so that it can be shared between three children?) and folding napkins.

Finally in this section, it is worth returning to the sustained attention and self-regulation that have been argued to characterise children’s engagement with the learning materials in the Montessori classroom.^2–4^ These are important parts of the complex cognitive construct of executive functions (EFs), which also include inhibition, working memory and planning. Put simply, EFs are the set of processes that allow us to control our thoughts and actions in order to engage in motivated, goal-directed behaviour. That EFs are critical for academic success is backed by a wealth of research evidence.^57–61^ Given this key role, EFs have become the target for a number of individually-administered interventions, full curricula, and add-ons to classroom curricula, such as CogMed (Pearson Education, Upper Saddle River, NJ), Tools of the Mind,^62^ PATHS (PATHS Training LLC, Seattle, WA), music, yoga and martial arts. A review study compared these, including Montessori education, and concluded that compared to interventions such as CogMed that solely target EFs, 'school curricula hold the greatest promise for accessibility to all and intervening early enough to get children on a positive trajectory from the start and affecting EFs most broadly'.^63^

## Conclusions

Montessori education has been in existence for over a hundred years. Such longevity could well be due, at least in part, to its adaptability.^6^ However, by its very nature, of course, greater adaptability means lower fidelity. This paper has discussed evidence that children may benefit cognitively and socially from Montessori education that is faithful to its creator’s principles, but it is less clear that adapted forms—which usually result in children spending less time engaged with self-chosen learning materials—are as effective. Nevertheless, studies suggest that the practical life materials can be usefully introduced into non-Montessori classrooms to support the development of young children’s fine motor skills and attention, and there is ample evidence from the wider educational literature that certain elements of the Montessori method—such as teaching early literacy through a phonic approach embedded in a rich language context, and providing a sensorial foundation for mathematics education—are effective. It has not been possible in this paper to give an exhaustive discussion of all the elements of Montessori education that might be beneficial, for example the lack of extrinsic rewards, the reduced emphasis on academic testing and lack of competition between pupils, the 3-year age-banding that fosters cross-age tutoring, or the presence of a trained teacher in the early years classroom.

Where does this leave Montessori education more than 100 years after its birth, and more than 60 years after the death of its creator? As others have noted, Montessori was a scientist who truly valued the scientific method and would not have expected her educational method to remain static.^64^ Yet Montessori teachers often feel fear or uncertainty about being able to apply Montessori’s theories in new and innovative ways while still adhering to her underlying philosophical principles.^65^ Ultimately, only empirical research, undertaken by teachers and researchers working together, can be our guide, because the questions that need answering are empirical in nature. Neuroscientific research—using neuroimaging methods which were not available in Montessori’s day—might also play a guiding role. For example, Montessori was prescient in her views that adolescence was a special time in development where the individual required a specially-designed form of education to address their needs.^66^ Recent neuroimaging evidence points to adolescence as indeed being an important period for neural development, particularly for areas involved in executive functions and social cognition.^67,68^ Montessori did not fully develop her ideas for the education of 12–18-year olds during her lifetime, but it is an area where current Montessorians might be able to take over the reins. Although some Montessori schools take pupils up to the age of 18, they are few and far between, and to my knowledge there are no published evaluations of their effectiveness. Developing a Montessori education for this age group in conjunction with the best of our current knowledge of developmental cognitive neuroscience has the potential to make a very positive contribution.

Nor did Montessori consider using her method with the elderly. In the context of a rapidly aging population and increasing numbers of elderly adults with acquired cognitive impairments such as those that result from Alzheimer’s disease,^69^ it is interesting to note that the Montessori method is now being adapted for use with dementia patients, with the aim of improving functioning in activities of daily living, such as feeding, and in cognition. There is strong evidence for a reduction in difficulties with eating, weak evidence for benefits on cognition, and mixed evidence for benefits on constructive engagement and positive affect.^70^ However, the quality of studies varies across domains; those evaluating effects on cognition have been of rather poor quality so far, and they have not yet examined whether there might be long-term effects. Nevertheless, given the challenges to developing successful medication for patients with Alzheimer’s disease despite a detailed knowledge of changes in their neurobiology, it would be sensible to continue the search for successful behavioural interventions alongside that for medical interventions.^71^ One method for delivering Montessori-based activities to the elderly is via inter-generational programmes, whereby older adults with dementia are supported in teaching Montessori-based lessons to preschool children. Benefits have been reported for the adults involved,^72^ but whether the children also benefit in particular ways from such inter-generational teaching has not been evaluated. Nor is it known whether a Montessori education in childhood or Montessori-based activities experienced in later life can protect the executive control circuits of the brain, as has been proposed for bilingualism.^73^ A lifespan approach to the evaluation of the Montessori method involving both behavioural and neuroimaging methods might be valuable.

In sum, there are many methodological challenges to carrying out good quality educational research, including good quality research on the Montessori method. Arguably the most obvious challenge to emerge from the literature reviewed here is the practical difficulty of randomly allocating pupils to Montessori and non-Montessori schools in order to compare outcomes. The majority of studies have relied instead on trying to match pupils and teachers in Montessori and non-Montessori schools on a number of different variables, with the concomitant danger that unidentified factors have contributed to any difference in outcomes. Even if randomisation is achievable, studies need to be conducted on a large enough scale to not only allow generalisations to be made beyond the particular schools studied, but to also allow investigation of which children the Montessori method suits best. On a more optimistic note, recent experimental studies—whereby features of existing Montessori classrooms are manipulated in some way, or features of the Montessori method are added to non-Montessori classrooms—hold promise for investigating the effectiveness of particular elements of the Montessori method. The evidence base can be strengthened yet further by drawing on research of educational interventions with which it shares certain elements, and by drawing on related research in the science of learning. National and regional education systems are beset by regular swings of the pendulum, for example towards and away from phonics,^74^ and towards and away from children working individually.^75^ This means that elements of the Montessori method will sometimes be in vogue and sometimes not. It is therefore particularly important that Montessori teachers understand the evidence base that supports, or does not support, their pedagogy.
